# Stricture of the Urethra

**Published:** 1915-08

**Authors:** Henry H. Morton


### Stricture of the Urethra.

By HENRY H. MORTON, M. D.

Clinical Professor of Genito-Urinary Diseases in the Long Island College Hospital; Genito-Urinary Surgeon to Long Island College and Kings County Hospitals, and the Polhemus Memorial Clinic, etc., Brooklyn, N. Y.

Organic stricture is a ring of fibrous tissue surrounding the urethra which interferes with its dilatability, later contracting and causing a narrowing of the urethral canal.

Its causes are:

1. Gonorrhea.
2. Traumatism causing a rupture of the urethra.

Clinically two varieties are recognized:

1. Soft or recent, in which the infiltration is recent and the small round cells are not organized.
2. Hard or organized, in which cicatricial changes have taken place.

Forms of strictures are:

1. Linear, which consists of a fine band of fibres.
2. Annular, in which the band is broader and firmer.
3. Tortuous, which is composed of heavy irregular masses of scar tissue causing more or less distortion and narrowing of the canal.

As to number, gonorrheal are usually multiple, while traumatic are usually single at the site of rupture.

The changes behind the stricture are very important, a pouch is formed which retains a drop of urine, which decomposes, irritates the mucous membrane and causes a gleety discharge. Prolonged inflammation leads to ulceration, which if small forms an abscess, opens externally and leads to fistula, or if large to extravasation of urine.

The walls of the bladder hypertrophy and the muscular fibers lose their elasticity which is followed by atony. The urine accumulates, decomposes and sets up a cystitis. Back pressure on the kidneys leads to dilation of the ureters and pelvis of the kidney. Infection follows and pyelitis or pyleonephritis develops and death follows.

The most constant symptoms are:

1. Frequency of urination due to congestion and irritability of posterior urethra. Later due to cystitis. In the later stages the bladder is distended and the overflow keeps dribbling away.
2. Dribbling after urination due to retention of a few drops of urine in the pouch behind the stricture.
3. Distorted and smaller stream.

4. Gleety discharge from meatus and shreds in urine.
5. Retention of urine due to congestion and swelling of mucous membrane following exposure to cold, alcohol, or sexual indulgence.
6. Pain in urethra is an inconstant symptom.
7. Impotence with feeble erection or premature ejaculation due to irritable posterior urethra.

Diagnosis is made by feeling irregularities in canal with flexible bulbous bougie. Meatotomy should be done if meatus is small so a fair-sized bougie may be used.

---

**A Leper Discharged From the Mercedes Asylum After Six Years Incarceration.** Elias Rojas, Gaceta Medica of Costa Rica, Jan. 15, 1915.

This case furnishes a bone of contention, with the pathologists arrayed on one side and the physicians in general practice on the other. Dr. Rojas originally made the diagnosis of anaesthetic leprosy, found the bacillus of Hansen in the nasal secretions and sent the patient to the hospital. There he attended him for six years, at which time the laboratory men stepped in and, their experiments proving negative, declared that the man was not a leper. The patient was then discharged.

Dr. Rojas states that the laboratory is a great assistance to the physician when the results are positive but loses its value when negative results are obtained. That the physician should rely primarily on the visible symptoms, palpable and positive which the patient presents for his observation. That in medical science no number of negative signs can ever prevail against positive signs. He calls attention to the two common forms of the disease: tubercular and anaesthetic—the former active, exhibiting, nearly always, the bacillus and giving a positive Wassermann—the latter seldom revealing the bacillus and furnishing a negative Wassermann. He further calls attention to the fact that the bacillus of Hansen may disappear from a given case after causing wide-spread lesions. He concludes that the release of this patient is most unfortunate, the evil result of which cannot be calculated. That it may have the further effect of bringing about the discharge of other cases of anaesthetic leprosy in which the bacillus of Hansen is not present, is to be deplored.—W. W. Q.

---

**Oesophageal Stenosis Due to Typhoid.** H. Horace Grant of Louisville, Pediatrics, June, reports a case in a boy of 13 seen two months after the disease, and an earlier case not described in detail but recovering after dilatation. The first case was treated by gastrostomy.

